# Characterization of Brazilian hospital admissions due to cardiovascular diseases: a longitudinal study

**DOI:** 10.1186/s12872-020-01588-w

**Published:** 2020-06-29

**Authors:** Luiza Gabriela de Araújo Fonseca, Illia Nadinne Dantas Florentino Lima, Lucien Peroni Gualdi

**Affiliations:** Programa de Pós Graduação em Ciências da Reabilitação, Faculdade de Ciências da Saúde do Trairi (FACISA)/Universidade Federal do Rio Grande do Norte (UFRN), Rua Vila do Trairi S/N, CEP, Santa Cruz, Rio Grande do Norte 59200-000 Brazil

**Keywords:** Health services research, Cardiovascular diseases, Hospitalization

## Abstract

**Background:**

Cardiovascular diseases (CVD) are the main cause of death and comorbidities worldwide. It is estimated that three quarters of all deaths related to CVD occur in low and middle income countries such as Brazil. Furthermore, it is estimated that emerging countries will present the highest worldwide prevalence of such diseases by 2050. In view of the above, this study aims to characterize Brazilian hospital admission distribution classified by the ICD-10 in adults between 2008 and 2017 in Brazil.

**Methods:**

This is a longitudinal descriptive study in which all data regarding hospital admissions registered in the Brazilian Hospital Information System of *“Sistema Único de Saúde”* (SIH/SUS) due to cardiovascular diseases (ICD-10) were included. All admissions from private or public services linked to the SUS from 2008 and 2017 were evaluated. The following variables were collected: number of hospital admissions, place of hospitalization classified by the ICD-10 and mortality rate at the federal level and according to regions. Absolute values and frequency of hospital admissions were grouped according to sex, age and living region as well as the number of deaths. The extracted data was stored in a Microsoft Excel 2013 program spreadsheet. Statistical analysis was performed by GraphPad Prism version 5.0 software.

**Results:**

There was a total of 11,345,821 hospital admissions due to CVD registered between 2008 and 2017. Individuals from 50 to 79 years old were the most affected. Heart failure (21.3%), other ischemic heart diseases (13.3%) and stroke (11.4%) were responsible for almost half of the hospital admissions associated to CVD. The number of registered deaths caused by any CVD was 867,838 and the national mortality rate was 7.82.

**Conclusion:**

CVD were responsible for around 10% of all hospital admissions in Brazil between 2008 and 2017. Moreover, it was possible to observe a decrease in hospital admissions as well as mortality rate over time after implementing governmental strategies to prevent cardiovascular diseases.

## Background

According to the world health organization (WHO) (2017) cardiovascular diseases (CVD) are the main cause of death worldwide. Thus, more individuals die annually due to these diseases than from any other cause. It is also estimated that three quarters of all deaths related to cardiovascular diseases occur in low and middle income countries such as Brazil. Studies have also shown that 37% of the 17 million premature deaths associated to non-communicable diseases (NCD) are caused by CVD [1].

The epidemiological transition process caused by aging, urbanization, social and economic changes as well as globalization have impacted the way of living, working and eating in the Brazilian population. Behavioral risk factors or those subject to interventions such as smoking, unhealthy eating, physical inactivity, alcohol and consumption of other drugs are potentiated by socioeconomic, cultural and environmental conditioning factors. Almost half of the Brazilian adults (≥ 18 years old) living in state capitals in 2011 reported weight excess (48.5%), 17% reported abusive alcohol consumption, 20% reported to consume insufficient quantities of fruits and vegetables and 14% were physically inactive [2].

According to the health ministry, the impact of risk and protection factors on NCD mortality may be perceived by the number of deaths attributed or preventable by each factor. An example to explain such an affirmation may be arterial hypertension which is the main risk factor for CVD and causes around 7.5 million deaths/year (12.8% of all deaths). On the other hand, regular physical activity and appropriate fruit and vegetable consumption reduces CVD risk.

Non-communicable diseases, which only represented 12% in the 1930s, have shown a considerable increase [3], corresponding to 73.9% of deaths in Brazil in 2010. From this total, 80.1% occurred due to cardiovascular diseases, cancer, chronic respiratory diseases and diabetes [2].

In addition, individuals with a cardiovascular disease are more likely to be hospitalized, present a higher risk of functional impairment, drug-related adverse events and higher prevalence of associated comorbidities [4]. It was estimated that 70% of healthcare spending was associated to NCD in Brazil in 2008. Moreover, inpatient care due to cardiovascular diseases cost R$ 1,183,712.23 totaling 561,350 hospital admissions in the first 6 months of 2018 [5].

In the coming decades, the so-called emerging countries which are unprepared for such social demand, together with industrialized countries will concentrate even higher numbers of deaths due to NCD. Even more, it is estimated that emerging countries will present the highest worldwide prevalence of such diseases by 2050 [6]. This increase will create several challenges for local, regional and national health managers. In fact, chronic diseases are very costly to public health systems such as the Brazilian *Sistema Único de Saúde*, especially when they are not properly prevented and managed.

Thus, it is worth highlighting the necessity to investigate the distribution of Brazilian hospital admissions according to cardiovascular disease profiles aiming to elaborate new preventive strategies to minimize hospital costs.

In view of the above, this study aims to characterize Brazilian hospital admissions distribution classified by the ICD-10 in adults between 2008 and 2017, as well as to analyze the incidence of hospital admissions, the regional distribution, and to observe the temporal trend of hospital admissions and mortality rate due to cardiovascular diseases in Brazil.

## Methods

### Study design

This is a longitudinal descriptive study in which all data regarding hospital admissions registered in the Brazilian Hospital Information System of the *“Sistema Único de Saúde”* (*SIH/SUS*) due to cardiovascular diseases (ICD-10) were included. All admissions from private or public services linked to the *SUS* from 2008 and 2017 were evaluated.

### Data extraction

Data were collected from the *SIH/SUS* provided by the Health Surveillance Bureau of the Brazilian Ministry of Health through its open access webpage available at the Department of Informatics of the Unified Health System (*DATASUS*). The following variables were collected: number of hospital admissions, place of hospitalization classified by the ICD-10, number of hospital admissions between 2008 and 2017 at the federal level and according to regions, absolute values and frequency by age group, sex and mortality rate.

The absolute values and frequency of hospital admissions were grouped according to sex, age and living region as well as the number of deaths recorded between 2008 and 2017. All data were provided by the hospital information system (*SIH/SUS*) from an online platform (link: http://datasus.saude.gov.br/) and were collected from July to December, 2018.

### Data analysis

Extracted data were stored in a Microsoft Excel 2013 program spreadsheet. Statistical analysis was performed by the GraphPad Prism version 5.0 software program. Data normality was assessed by the Shapiro-Wilk test. Comparisons among the groups were performed by the Kruskal-Wallis test and Dunn’s Multiple Comparison post hoc test. A *p* value < 0.05 was considered significant.

## Results

A total of 112,265,103 hospital admissions were registered in the *SIH/SUS* system between 2008 and 2017. From this total, 11,345,821 occurred due to CVD. Heart failure (21.3%), other ischemic heart diseases (13.3%) and stroke (11.4%) were responsible for almost half of the hospital admissions associated to CVD (Table 1).

**Table 1:** Distribution of hospitalizations by place of hospitalization, classified of ICD-10 circulatory system diseases, Brazil, 2008–2017

Description	Sex		Frequency %	
	Male	Female		
Acute rheumatic fever	20,882	21,838	42,720	0.4
Chronic rheumatic heart diseases	33,718	45,248	78,966	0.7
Essential (primary) hypertension	334,594	484,697	819,291	7.2
Other hypertensive diseases	120,232	175,237	295,469	2.6
Acute myocardial infarction	554,051	319,162	873,213	7.7
**Other diseases ischemic acute the heart**	**885**,**643**	**621**,**154**	**1**,**506**,**797**	**13.3**
Pulmonary embolism	24,569	37,834	62,403	0.6
Conduction disorders and cardiac arrhythmias	291,565	278,730	570,295	5.0
**Cardiac insufficiency**	**1**,**237**,**943**	**1**,**176,789**	**2**,**414**,**732**	**21.3**
Other heart diseases	175,048	186,907	361,955	3.2
Intracerebral haemorrhage	162,984	154,046	317,030	2.8
Cerebral infarction	82,211	75,945	158,156	1.4
**Stroke, Not Specified as Hemorrhagic or Ischemic**	**667,867**	**623,021**	**1**,**290**,**888**	**11.4**
Other Cerebrovascular Diseases	80,379	79,812	160,191	1.4
Atherosclerosis	87,427	73,258	160,685	1.4
Other Peripheral Vascular Diseases	44,873	30,031	74,904	0.7
Embolism and Arterial Thrombosis	104,270	78,370	182,640	1.6
Other Disorders of Arteries and Arterioles	215,372	134,821	350,193	3.1
Phlebitis and Thrombophlebitis	148,349	232,405	380,754	3.4
Lower Limb Varicose Veins	191,970	642,773	834,743	7.4
Hemorrhoids	125,327	165,117	290,444	2.6
Other and unspecified Circulatory Tract Disorders	80,357	39,052	119,409	1.1
Total	5,669,631	5,676,247	11,345,878	100.0

Font: Ministry of Health - SUS Hospital Information System (SIH / SUS)

### CVD incidence according to Brazilian region

**Fig. 1:**
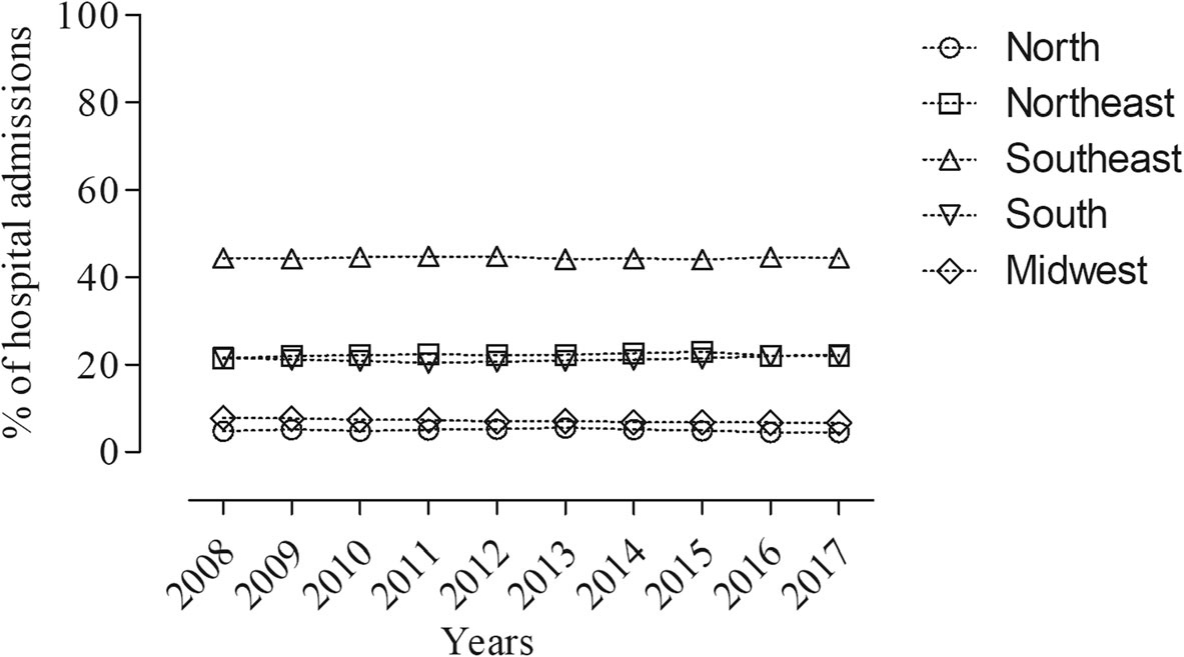
Relative frequency of hospital admissions caused by cardiovascular diseases according to geographic region from 2008 to 2017. Font: Ministry of Health - SUS Hospital Information System (SIH / SUS)

When we grouped the incidence of CVD according to regions it was found that 44.4% (*n* = 5,041,034) of hospital admissions occurred in the Southeast region (states of Espírito Santo, Minas Gerais, Rio de Janeiro and São Paulo) region which represents a 10 times higher prevalence when compared to the Northern region. This is followed by the Northeast region (states of Alagoas, Ceará, Maranhão, Paraíba, Pernambuco, Piauí, Rio Grande do Norte and Sergipe) (*n* = 2,521,200), the South region (states of Paraná, Rio Grande do Sul and Santa Catarina) (n = 2,411,420), the Midwest region (states of Goiás, Mato Grosso and Mato Grosso do Sul and Distrito Federal) (*n* = 807,937) and he Northern region (states of Acre, Amapá, Amazonas, Pará, Rondônia, Roraima and Tocantins) (*n* = 564,230). Figure 1 shows the percentage described annually during this period.

### CVD incidence according to age

Table 2 shows that most of the hospital admissions caused by impairment to the cardiovascular system occurs in subjects aged between 50 and 79 years, which is similar in all Brazilian regions. Statistical differences were found when we compared the 20–29 age group to 50–59, 60–69 and 70–79 age groups as well as between the 30–39 and 70–79 age groups (*p* < 0.0001).

**Table 2:** Absolute and relative frequency of hospitalizations caused by cardiovascular diseases regarding geographic region and age groups, between 2008 and 2017

Age range			Regions			p value
	North^a^	Northeast^b^	Southeast^c^	South^d^	Midwest^e^	
20–29	26,469 (4.7)^bc^	97,995 (3.9)^a e^	143,315 (2.8)^a d e^	55,379 (2.3) ^c^	27,854 (3.4)^bc^	< 0.0001
30–39	43,693 (7.7)^bcd^	169,859 (6.7)^a^	313,016 (6.2)^a d e^	122,429 (5.1)^ac^	56,706 (7.0)^bc^	< 0.0001
40–49	67,440 (12.0)^bcd^	283,714 (11.3)^a^	615,446 (12.2)^a^	278,671 (11.6)^ae^	104,216 (12.9)^c^	< 0.0001
50–59	100,647 (17.8)^bcd^	433,012 (17.2)^ac^	1,051,132 (20.9)^abe^	493,168 (20.5)^ae^	161,939 (20.0)^cd^	< 0.0001
60–69	120,098 (21.3)^bcd^	546,823 (21.7)^ac^	1,188,522 (23.6)^abe^	597,849 (24.8)^ae^	184,022 (22.8)^cd^	< 0.0001
70–79	112,225 (19.9)^bcd^	526,09 (20.9)^a^	1,005,225 (19.9)^ae^	520,615 (21.6)^a^	160,761 (19.9)^c^	< 0.0001
≥80	72,334 (12.8)^bcd^	387,655 (15.4)^ae^	635,092 (12.6)^a d e^	303,782 (12.6)^ac^	90,284 (11.2)^bc^	< 0.0001
p valor	< 0.0001	< 0.0001	< 0.0001	< 0.0001	< 0.0001	

Legend: Absolute frequency (%). ^a^North; ^b^Northeast; ^c^Southeast; ^d^South and ^e^Midwest; expressing statistically significant differences among regions

Font: Ministry of Health - SUS Hospital Information System (SIH / SUS)

**Fig. 2:**
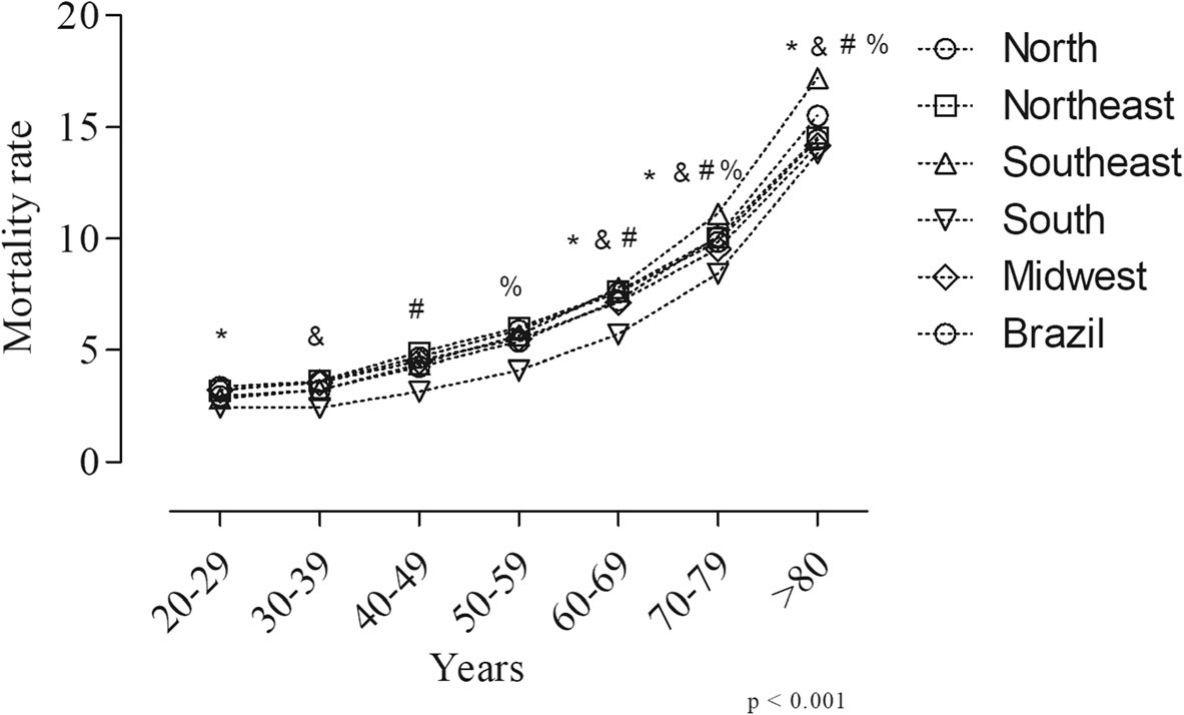
Hospital admissions’ mortality rate according to geographic region and age group between 2008 and 2017; *significant difference between 20 and 29 and 60,69, 70–79 and > 80 age groups; ^&^significant difference between 30 and 39 and 60,69, 70–79 and > 80 age groups; ^#^significant difference between 40 and 49 and 60,69, 70–79 and > 80 age groups; ^%^significant difference between 50 and 59 and 70–79 and > 80 age groups; *p* value < 0.001. Font: Ministry of Health - SUS Hospital Information System (SIH / SUS)

The number of registered deaths caused by any CVD between 2008 and 2017 was 867,838. The mortality rate was determined by the ratio of the number of deaths and the number of hospital admissions approved and authorized (which were computed as hospital admissions) between 2008 and 2017, and multiplied by 100. Thus, the national mortality rate was 7.82. This number was higher than the national average in the Southeast and Northeast regions, and increased according to age group (Fig. 2). The mortality rate was significantly lower in the 20–29 and 30–39 groups in comparison to the 60–69, 70–79 and > 80 groups (*p* < 0.001). The 40–49 age group showed a significantly lower mortality rate when compared to the 60–69, 70–79 and > 80 groups (p < 0.001), while the 50–59 age group showed a significant difference when compared to the 70–79 and > 80 groups (p < 0.001). However, no significant differences were found among mortality rates when we compared geographic regions (*p* > 0.05).

### Longitudinal variation of hospital admissions due to CVD

When hospital admissions were accessed according to annual variation we, found that there were variable changes along the years; however, they showed a decrease in growth amplitude despite the annual increase.

**Fig. 3:**
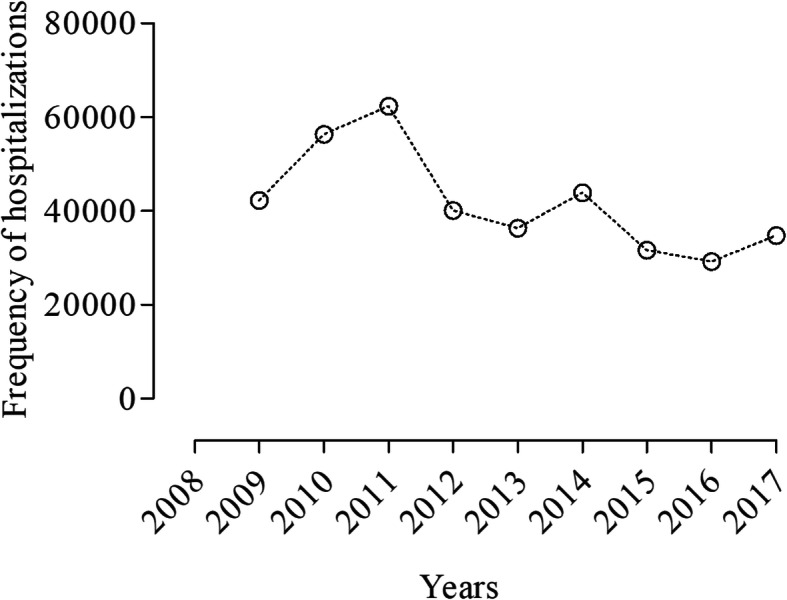
Hospital admissions’ incidence caused by cardiovascular diseases from 2008 to 2017 in Brazil. Font: Ministry of Health - SUS Hospital Information System (SIH / SUS)

Thus, an increased variation of 42,252 hospital admissions was found between 2008 and 2009, followed by 2009 to 2010 (*n* = 14,073) and 2010 to 2011 (*n* = 5997) (*p* > 0.05). We also found declined incidence between 2011 and 2012 (*n* = 22,186) and 2012 to 2013 (*n* = 3789), followed by an increase between 2013 and 2014 (*n* = 7557), and another decline between 2014 and 2015 (*n* = 12,271) and 2015 and 2016 (2381) (*p* > 0.05), as shown in Fig. 3.

Moreover, an increase of 62,322 hospital admissions was observed between 2008 and 2011 (*p* > 0.05). This increase was of 3.85% in 2009, followed by 5.13% in 2010 and 6.58% in 2011 in comparison to 2008 (p > 0.05). We found a reduction of 1.5% when we assessed 2012, in comparison to 2008 (p > 0.05). This decrease was followed by an increase of 3.31% in 2013, 4.0% in 2014, 2.88% in 2015, 2.66% in 2016 and 3.16% in 2017 (p > 0.05). Although we have shown different delta variations along the study period, no significant difference was found when we compared the total number of hospitalizations among the years.

## Discussion

In this study heart failure was responsible for the highest number of hospital admissions among the main causes classified by the ICD-10 in all Brazilian regions between 2008 and 2017. Such prevalence may be explained by the improvement of ischemic disease care and the treatment of heart failure with drugs and devices such as pacemakers and artificial ventricles, as well as population aging which results in an increase in the costs related to hospital admissions for the health systems [7].

Population aging and increased prevalence of cardiovascular risk factors, such as hypertension and diabetes, have been the main factors responsible for the increasing impact of CVD in Brazil in the last decades [8]. According to WHO, the most prevalent CVD are ischemic heart disease and cerebrovascular disease, which corroborates with the results found herein. However, such health conditions show common risk factors, as well as potentially changeable factors which may be changed by healthier lifestyles [9].

Nevertheless, the literature highlights the existence of important regional differences corroborating with the findings of our study. The pyramid that represents the Northern region still preserves the characteristics of a young population, as well as the South which is marked by a typical demographic transition process [3]. It is also important to highlight the expressive demographic, social and economic heterogeneity existing in Brazil which is reflected in different mortality and morbidity patterns among the study results. Such differences demand actions which involve local managers and are adequate to the reality found in each state.

Although CVD may occur in all ages, its incidence increases as age increases or in those who are 75 years old or more. Thus, the longer the longevity, the higher the likelihood of being affected by such diseases [10]. Such an affirmation corroborates with our results, as we found that higher age groups equated to higher hospital admission incidence.

The greater prevalence of CVD in individuals aged 50 years or more seems to be related to the increase in life expectancy in the Brazilian population, which has been showing positive changes in the last decades. It is estimated that 200 thousand people turns 60 years old each year in the Brazilian population, which generates an important demand on health systems as the number of hospital admissions and health care increase [3].

On the other hand, life expectancy at birth has also shown a progressive increase in the last decades. Life expectancy in 1980 was 62.6 years, and this number increased to 75.8 years in 2016. However, life expectancy differs among social stratum, region and Brazilian states [11]. Such conditions may explain the study results, as we found regional differences among age groups in the number of hospital admissions.

The Brazilian Ministry of Health launched a national action plan in 2011, and established several directions for federal investments, among which the priorities were to focus on the commitment to control chronic non-communicable diseases and their risk factors. The planned goals are similar to global goals and refer to mortality reduction by chronic non-communicable diseases by reducing the use of tobacco, alcohol, salt and obesity [12]. Other Brazilian goals include increasing physical activity and the consumption of fruits and vegetables [13]. The Brazilian government adopted all global goals.

In this context, it is worth highlighting a reduction of hospital admissions from 2011 to currently, as shown in several studies. It is also important to note that all Brazilian regions have suffered socioeconomic changes in the last 30 years. However, we may consider that the changes occurred at different velocities [14] in order to explain the differences among regions.

Brazil is going through a fast demographic transition process in which we can observe several changes regarding population increase and age group distribution. According to the Brazilian Institute of Geography and Statistics (IBGE) [15], there are more than 20 million people aged 60 years or more in Brazil currently which represents 11% of the total population. This profile change in association to increased life expectancy leads to an increase in health costs as well as social security in the Brazilian population. Studies have shown that whichever the indicators are observed in health quality evaluations for the older adult population, they point to a higher use of health services and costs when compared to the younger population [16].

This study has some limitations such as disease diagnosis being performed by the ICD code. This classification is not suitable if little or no information about the patient is available, as symptoms can be caused by several different conditions. It is important to highlight that due to the characteristics of our population (hospitalized subjects) the ICD diagnosis was made after several visits, which reduces the changes of wrong diagnoses. Moreover, echocardiographic measurements, angiographic imaging and clinical signs/markers may be used to confirm diagnosis, however no information about such data are available in the *DATASUS* system. Finally, the ICD classification is the most important classification as a coding system in medical databases to support research and public health reports, and is most used by physicians. Moreover, due to continuous update of *DATASUS* system we are not able to calculate the accurate proportion of public and private services from study data as such information was not extracted from the system during data extraction. However, after last system update the percentage of private services was 46.44%, public services 32.04% and unknown/ignored 21.52%.

## Conclusion

The Brazilian population from 50 to 79 years showed higher hospital admission frequency due to cardiovascular diseases. A decrease in the number of hospital admission over the years was also observed after implementation of governmental programs which prioritize life quality, physical activity and preventive healthcare, and which reflects equally national decreasing levels in hospital admissions.

## Data Availability

The datasets analyzed during the current study are available in the Brazilian system of Hospital Information of *“Sistema Único de Saúde”* repository, link: http://datasus.saude.gov.br/.

## Abbreviations

CVD: Cardiovascular diseases
DATASUS: Department of Informatics of the Unified Health System
ICD: international classification of diseases
NCD: non-communicable diseases
SIH/SUS: hospital information system/“*sistema único de saúde”*
WHO: world health organization
